# Concurrent validity and agreement of the HerniaCare Lab device for abdominal wall strength assessment

**DOI:** 10.1007/s10029-025-03580-9

**Published:** 2026-03-10

**Authors:** José Luis Gil Delgado, Carlos Rangel Cascajosa, Alejandro Sánchez Arteaga, Luis Tallón Aguilar, Borja Sañudo Corrales

**Affiliations:** 1 Department of Physical Education and Sports, University of Seville, Seville, Spain; 2 Department of General Surgery, Hospital Universitario Virgen del Rocío, Avda. Manuel Siurot S/N, Seville, 41013 Spain

**Keywords:** Abdominal wall hernia, Muscle strength assessment, Device validation, Hand-held dynamometry

## Abstract

**Purpose:**

Currently, no standardized, low-cost, and portable method is available for assessing abdominal wall strength in patients with incisional hernias, addressing the limitations of traditional dynamometry.

**Methods:**

This cross-sectional validation study compared the HerniaCare Lab device performance with the Activforce 2 hand-held dynamometer in92 adults diagnosed with abdominal wall hernias. Isometric trunk flexion strength was measured under identical conditions, and agreement between devices was analyzed using non-parametric tests, correlation, and concordance statistics.

**Results:**

The HerniaCare Lab showed systematically higher strength values than the Activforce 2 (mean difference = + 22.3 N, p < 0.001) but demonstrated a very strong positive correlation (ρ = 0.95, p < 0.001) and good concordance (CCC = 0.89). Bland–Altman analysis revealed a mean bias of + 24.9 N with 95% limits of agreement from − 17.7 to+ 67.5 N, and a slight proportional bias at higher force levels.

**Conclusion:**

Despite predictable overestimation, the HerniaCare Lab exhibits strong concurrent validity and good agreement with an established reference device, supporting its potential clinical utility for objective, reproducible, and accessible assessment of abdominal wall strength in surgical populations.

## Introduction

Abdominal wall hernias (AWH) represent one of the most common long-term complications after abdominal surgery, with incidence rates ranging from 11% in laparoscopic procedures to 23% in open abdominal surgery [1, 2]. These abdominal wall defects impose substantial clinical burden, leading to chronic pain in 65% of patients and significantly reduced quality of life [3, 4]. Recent evidence demonstrates that objective preoperative strength assessment correlates significantly with postoperative complications and length of hospital stay in abdominal wall reconstruction patients [5].

Despite this clinical relevance, standardized methods for evaluating abdominal wall strength remain limited. Traditional approaches rely on subjective physical examination and patient-reported outcomes, which lack precision and reproducibility [6]. While isokinetic dynamometry demonstrates excellent reliability (ICC = 0.92–0.97), high costs (50,000) and complex operational requirements preclude routine clinical use [7]. Hand-held dynamometry offers a portable alternative but suffers from examiner-dependent variability (coefficient of variation 8–15%) and inconsistent positioning protocols [8].

The HerniaCare Lab device was developed to address these limitations through a low-cost, portable, and standardized system for isometric abdominal wall strength assessment. By incorporating stable mechanical structure, controlled positioning, and digital dynamometry, the device aims to minimize examiner influence while maximizing reproducibility. The Activforce 2 hand-held dynamometer, previously validated for musculoskeletal strength testing with excellent test-retest reliability (ICC = 0.95–0.99), serves as an appropriate reference standard [9].

This study evaluates the concurrent validity and agreement of the HerniaCare Lab device against the Activforce 2 dynamometer in patients with incisional hernias. Establishing the validity of this innovative assessment tool represents a critical step toward its integration into clinical practice for improved patient selection, surgical planning, and rehabilitation monitoring in abdominal wall surgery.

## Methodology

### Study design

This was a cross-sectional validation study conducted over five consecutive data collection sessions at the outpatient surgical consultation of XXX. The aim was to assess the concurrent validity of a newly developed abdominal wall strength assessment device (HerniaCare Lab) by comparing its performance with a previously validated hand-held dynamometer (Activforce 2). All measurements were performed in a standardized clinical setting using the same testing protocol for both devices.

### Participants

A total of 92 adults (50 men and 42 women) diagnosed with AWH participated in this validation study. The mean age of the sample was 57.3 ± 15.2 years, with an average body mass of 76.2 ± 15.4 kg, height of 1.68 ± 0.09 m, and body mass index (BMI) of 27.2 ± 4.6 kg/m² (Table 1).

**Table 1:** Baseline characteristics of the study participants

	Age	Weight	Height	BMI	HerniaCare Lab	Activforce 2
N	92	93	93	93	93	93
Missing	1	0	0	0	0	0
Mean	57.1	76.3	1.67	27.4	176	151
Sd	15.9	16.2	0.105	4.92	72.1	64.5
Shapiro-Wilk W	0.977	0.979	0.980	0.984	0.942	0.950
Shapiro-Wilk p	0.098	0.137	0.166	0.298	< 0.001***	0.001**

Notes. Sd: standard deviation; BMI: body mass index;

* Significance *p* < 0.05;** Significance *p* < 0.01;*** Significance *p* < 0.001

Inclusion criteria comprised adult patients (≥ 18 years) with a confirmed diagnosis of abdominal wall hernia (AWH), classified as W2 or W3 according to the European Hernia Society (EHS) classification system, and an American Society of Anesthesiologists (ASA) physical status score below IV.

Exclusion criteria included W1 incisional hernias, lateral or parastomal hernias, and complicated presentations such as incarceration or strangulation. Patients with severe systemic comorbidities (ASA ≥ IV), advanced chronic diseases, or unresolved postoperative complications from previous hernia repairs were also excluded.

Hernias classified as EHS W1 were excluded because their small defect size is unlikely to produce a clinically relevant impairment in abdominal wall strength, potentially limiting the ability of the assessment to detect meaningful functional deficits.

Conversely, patients with EHS W4 (diameter ≥ 15 cm) defects were excluded because very large hernia widths may substantially alter abdominal wall biomechanics, compromise standardized force transmission during seated isometric trunk flexion, and increase variability in strength measurements. Restricting the sample to W2–W3 defects was therefore intended to enhance methodological homogeneity and ensure safe, reproducible testing conditions.

Additional exclusion factors were recent pregnancy (< 6 months), current substance or alcohol abuse, inability to participate in supervised testing sessions, language barriers without available translation, and failure to provide written informed consent prior to participation.

### Instruments

**Fig. 1:**
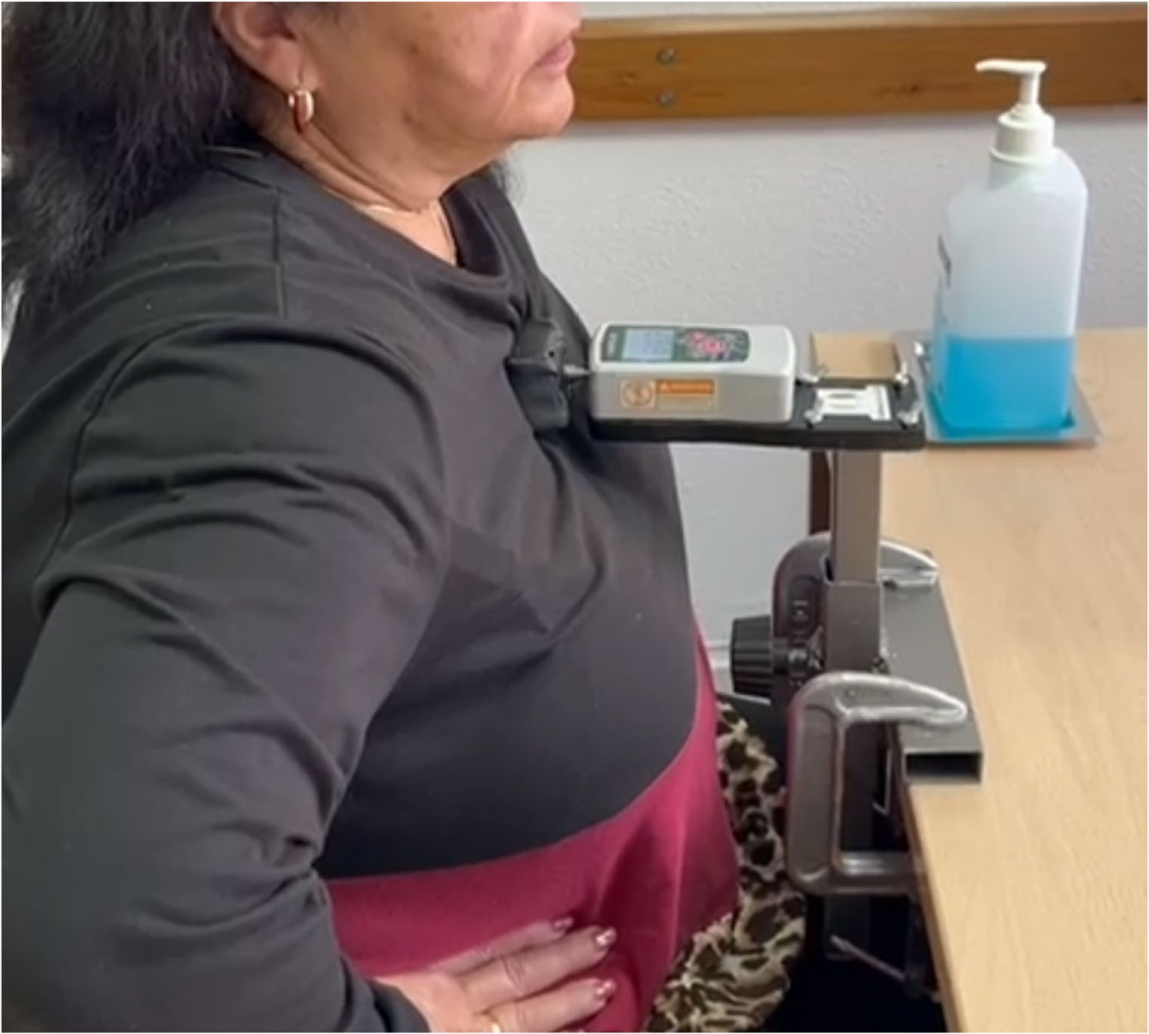
HerniaCare Lab device

The novel device HerniaCare Lab was used to assess abdominal wall muscle strength. This instrument was specifically designed to provide a portable, low-cost, and standardized method for evaluating isometric abdominal strength in patients with abdominal wall hernias. The system comprises four main components: (1) a vertical aluminum support structure with a stable base; (2) a height-adjustable horizontal aluminum arm; (3) a metallic clamp ensuring secure positioning of the dynamometer; and (4) a pressure sensor-based digital dynamometer (GOYOJO 500 N), which records the force applied by the patient in Newtons (N). The setup allows for reproducible positioning and measurement in a sagittal plane seated trunk flexion test, minimizing examiner interference (Fig. 1).

Measurements obtained with the HerniaCare Lab were recorded on the dynamometer’s built-in digital screen. The device has been designed for compatibility with a mobile application (currently in beta phase), which will enable clinicians to integrate strength data into personalized prehabilitation or rehabilitation training programs.

To validate the HerniaCare Lab, its measurements were compared against the Activforce 2, a commercially available digital hand-held dynamometer previously validated for musculoskeletal strength assessment [8, 10]. The Activforce 2 is equipped with a pressure-sensitive interface and Bluetooth connectivity, transmitting the force data (in Newtons) to its proprietary mobile application for real-time analysis and storage.

Both devices aimed to quantify peak isometric trunk flexion strength in Newtons (N) under standardized biomechanical conditions, allowing for concurrent validity assessment.

### Ethical considerations

This study was conducted in accordance with the ethical principles of the Declaration of Helsinki [11]. All participants provided informed consent prior to their inclusion in the study. According to institutional and national regulations, this observational study, involving only non-invasive isometric strength measurements performed during routine outpatient clinical assessment and without any modification of standard patient management, did not require formal review by an ethics committee. This exemption was formally confirmed by the institutional review body prior to study initiation.

### Testing procedures

**Fig. 2:**
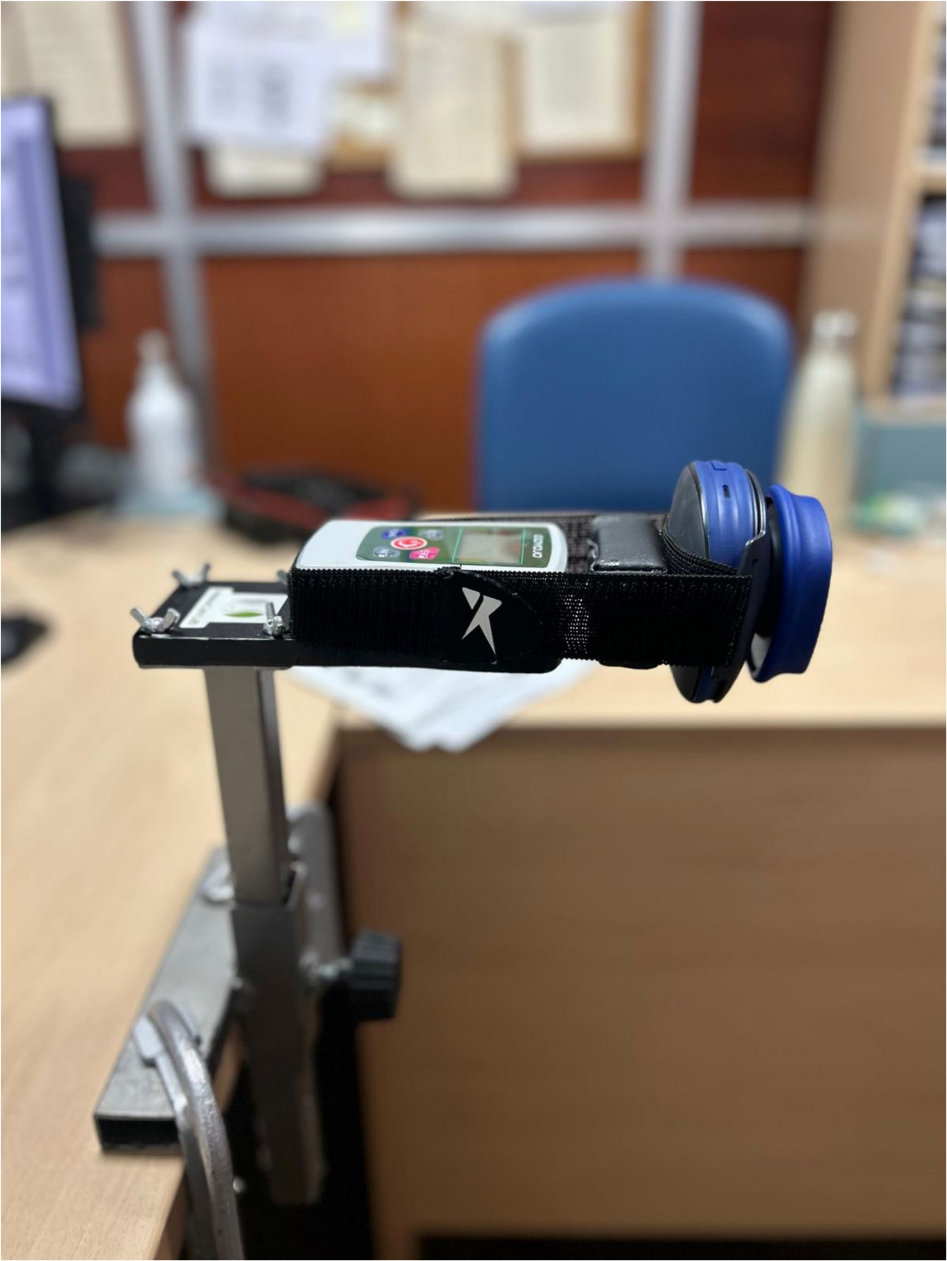
HerniaCare Lab and Activforce 2 set up

All trunk flexion strength assessments were conducted using a standardized protocol with participants positioned in a seated configuration. Participants were seated with their trunk and femur maintained at a 90° angle, with feet flat on the floor and back firmly supported against the chair. The Activforce 2 hand-held dynamometer was securely mounted and positioned anterior to the HerniaCare Lab device to enable simultaneous bilateral data acquisition from both instruments (Fig. 2).

Prior to testing, participants received standardized verbal instructions and completed one practice trial to ensure proper technique and familiarization with the testing procedure. Each participant then performed three maximal isometric trunk flexion contractions, with each contraction sustained for 4 s at maximum voluntary effort. Standardized verbal encouragement was provided during each trial to ensure maximal effort. A 60-second rest interval was implemented between consecutive trials to prevent muscular fatigue and maintain force output consistency.

Peak force values were identified from each of the three repetitions for both measurement devices. The final strength score for each participant was calculated as the arithmetic mean of the three peak force values from each device, providing a representative measure of maximal trunk flexion strength.

### Statistical analysis

All statistical analyses were performed using Jamovi software (version 2.6.21). Descriptive statistics were used to characterize the sample and to summarize the force values obtained from both measurement devices. Shapiro–Wilk test was applied to assess the normality of continuous variables.

As the paired differences did not meet the normality assumption, non-parametric methods were applied. Agreement between the HerniaCare Lab and the Activforce 2 was first evaluated using the Wilcoxon signed-rank test to detect any potential systematic bias between the instruments. The strength of association between measurements from both devices was assessed using the Spearman rank correlation coefficient (ρ). Following conventional interpretation, ρ values were classified as very weak (0.00–0.19), weak (0.20–0.39), moderate (0.40–0.59), strong (0.60–0.79), or very strong (0.80–1.00).

To assess concordance beyond correlation, Lin’s concordance correlation coefficient (CCC) was calculated. In this study, a conservative interpretative approach was used: CCC values greater than 0.80 were considered indicative of good concordance, while values exceeding 0.90 were interpreted as excellent concordance.

Additionally, Bland–Altman analysis was performed to estimate the mean bias and the 95% limits of agreement (LoA), defined as the mean difference ± 1.96 standard deviations of the differences. The presence of proportional bias was evaluated by regressing the differences on the means of the two devices. Statistical significance was set at *p* < 0.05 for all analyses.

## Results

A total of 93 participants completed the strength assessment protocol. Shapiro–Wilk tests indicated that force data from both devices deviated significantly from normality (HerniaCare Lab: W = 0.942, *p* < 0.001; Activforce 2: W = 0.950, *p* = 0.001), whereas anthropometric variables showed no significant departure from normal distribution (*p* > 0.05 in all cases).

The Wilcoxon signed-rank test revealed a statistically significant difference between the two devices (W = 4316, *p* < 0.001), with the HerniaCare Lab yielding higher values than the Activforce 2. The mean difference (bias) between devices was + 22.3 N in favor of the HerniaCare Lab, with a 95% confidence interval ranging from 18.5 to 26.4 N (Table 2).

**Table 2:** Wilcoxon Signed-Rank test results and mean bias between devices

							95% CI	
			Statistic	*p*	Mean difference	SE difference	Lower	Upper
**HerniaCare Lab**	**Activforce 2**	**Wilcoxon W**	4316	< 0.001***	22.3	2.25	18.5	26.4

Notes. CI: Confidence Interval;

* Significance *p* < 0.05;** Significance *p* < 0.01;*** Significance *p* < 0.001

A very strong positive relationship was observed between the two measurement tools, as indicated by the Spearman rank correlation coefficient (ρ = 0.95, *p* < 0.001) (Table 3).

**Table 3:** Spearman’s rank correlation coefficient between herniacare lab and activforce 2 measurements

		HerniaCare Lab	Activforce 2
**HerniaCare Lab**	Spearman’s rho	—	
	p-value	—	
**Activforce 2**	Spearman’s rho	0.952	—
	p-value	< 0.001***	—

* Significance *p* < 0.05;** Significance *p* < 0.01;*** Significance *p* < 0.001

To enhance the evaluation of the correlation between the devices, Lin’s concordance correlation coefficient (CCC) was computed, resulting in a value of 0.89, which suggests a good concordance between the HerniaCare Lab and the Activforce 2 (Table 4).

**Table 4:** Lin’s concordance correlation coefficient between herniacare lab and activforce 2

Comparison	CCC Value	Interpretation	Threshold Reference
HerniaCare Lab vs. Activforce 2	0.89	Good concordance	CCC > 0.80 = good; > 0.90 = excellent

Notes. CCC: concordance correlation coefficient

**Fig. 3:**
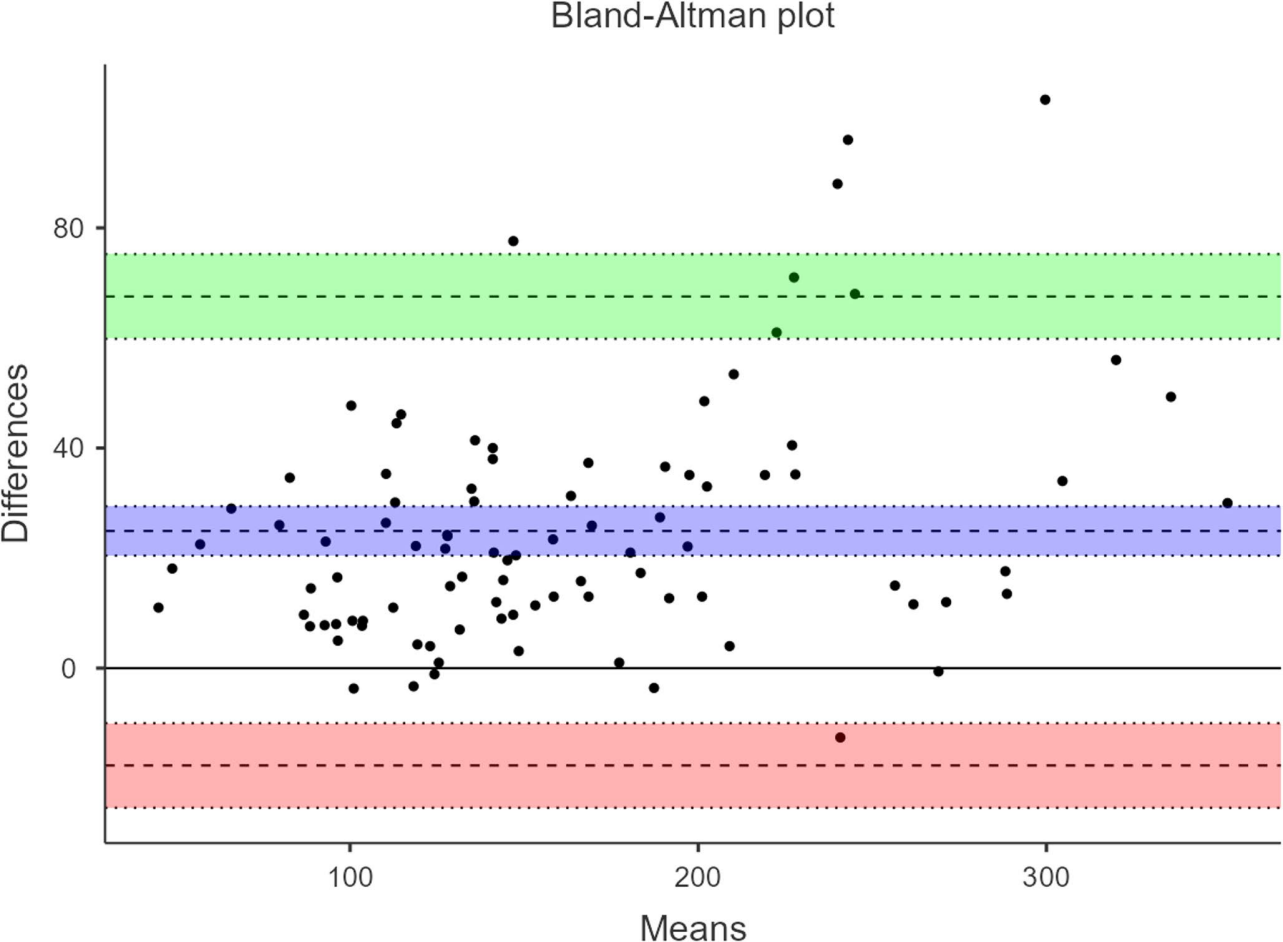
Bland–Altman Plot for Agreement Between HerniaCare Lab and Activforce 2

The Bland–Altman analysis showed a mean bias of + 24.9 N, with 95% limits of agreement ranging from − 17.7 to + 67.5 N. The regression analysis revealed a proportional bias (slope = 0.11), suggesting a slight overestimation by the HerniaCare Lab at higher force values. While the overall agreement was acceptable, the relatively wide limits of agreement indicate that discrepancies between instruments may be clinically relevant in certain individuals (Fig. 3).

## Discussion

This validation study demonstrates strong concurrent validity between the newly developed HerniaCare Lab device and the Activforce 2 dynamometer (reference standard) for measuring abdominal wall strength in patients with midline incisional hernias. The devices showed excellent rank-order agreement (ρ = 0.95, *p* < 0.001) and good overall concordance (CCC = 0.89), supporting the HerniaCare Lab’s potential as a valid clinical assessment tool. The observed correlation coefficient (ρ = 0.95) substantially exceeds values reported in previous dynamometer validation studies, which typically range from 0.37 to 0.82 for various muscle groups [12–14]. While a statistically significant systematic bias was observed, with the HerniaCare Lab consistently yielding higher force values, the strong correlation indicates both devices rank participants similarly in terms of strength—a critical requirement for clinical monitoring and progression tracking.

The HerniaCare Lab demonstrated consistent overestimation compared to the Activforce 2, with a systematic bias ranging from + 22.3 to + 24.9 N. This predictable deviation follows patterns observed in previous validation studies of hand-held dynamometers, where device-specific characteristics—including sensor placement, surface interface, and mechanical resistance—contribute to measurement variations [15]. The magnitude of bias observed in our study aligns with findings from Sahu et al., who reported mean absolute differences of 24–58 N between different dynamometer types when assessing knee extensor strength [14]. Similarly, systematic overestimation patterns in hand-held dynamometry across multiple muscle groups have been documented [8]. Importantly, the consistent nature of this bias enables potential calibration correction while preserving the device’s clinical utility for strength assessment and longitudinal monitoring.

Bland–Altman analysis revealed limits of agreement spanning − 17.7 to + 67.5 N, indicating that individual-level discrepancies may occasionally reach clinically meaningful magnitudes. The detected proportional bias demonstrates that the magnitude of overestimation by the HerniaCare Lab increases with higher force values, consistent with previous reports comparing handheld and isokinetic dynamometry, particularly at higher force outputs [16, 17].

In contrast to our findings, Jensen et al. (2016) reported no systematic bias when validating the Good Strength dynamometer for abdominal wall assessment in patients with incisional hernias [18], achieving excellent test-retest reliability (ICC = 0.91–0.99). However, their study utilized a different measurement approach and patient population, which may explain the divergent bias patterns. These findings emphasize that while the devices show strong group-level agreement, individual patient monitoring should maintain device consistency throughout the assessment period unless validated correction factors are applied.

Despite the identified limitations, the HerniaCare Lab offers distinct advantages that position it favorably for surgical populations. Its enhanced portability, cost-effectiveness, and potential for integration into digital prehabilitation platforms address key barriers to routine strength assessment in clinical practice. Unlike traditional dynamometers, the device’s standardized positioning design reduces inter-evaluator variability and minimizes evaluator influence, thereby enhancing measurement reproducibility—a critical factor in surgical settings [19, 20].

Future validation efforts should prioritize several key areas to establish comprehensive clinical utility. Test-retest reliability assessment is essential to determine measurement stability over time, particularly given the excellent reliability (ICC > 0.90) reported for similar abdominal strength measurement devices [18]. Responsiveness to clinically meaningful change must be established to enable effective monitoring of rehabilitation progress, building on the foundation of strong concurrent validity demonstrated in this study.

Predictive validity studies across diverse hernia populations—including ventral, umbilical, and parastomal hernias—will expand the device’s clinical applicability. Critical next steps include determining population-specific minimal clinically important differences and investigating the device’s integration into established surgical care pathways. Prospective studies examining whether HerniaCare Lab-guided prehabilitation protocols improve surgical outcomes would provide crucial evidence for widespread clinical adoption, particularly given the growing evidence for prehabilitation benefits in hernia surgery [21].

Additionally, exploring the clinical impact of incorporating objective strength assessments into preoperative risk stratification models could further validate the device’s utility in optimizing patient selection and perioperative management strategies. The device’s superior correlation compared to existing validation studies suggests strong potential for clinical implementation, pending confirmation of reliability and responsiveness characteristics.

## Conclusion

The HerniaCare Lab device demonstrates exceptionally strong concurrent validity (ρ = 0.95) with established dynamometry standards, exceeding correlation values reported in previous validation studies of similar devices. Despite systematic overestimation that requires consideration in clinical interpretation, the predictable nature of this bias and the device’s practical advantages make it a promising tool for strength evaluation in hernia surgery populations. The superior statistical performance compared to existing literature, combined with enhanced portability and standardization features, positions this device favorably for clinical adoption pending further validation of reliability and clinical utility outcomes.
